# *rac*-5-(1-Methyl­eth­yl)-2-sulfanylidene­imidazolidin-4-one

**DOI:** 10.1107/S1600536813008337

**Published:** 2013-04-05

**Authors:** Rosane de P. Castro, Fernando C. Macedo, Tiago O. Brito, Angelo de Fátima, José R. Sabino

**Affiliations:** a Instituto de Física – UFG, Caixa Postal 131, 74001-970 Goiânia, GO, Brazil; b Departamento de Química – UEL, Caixa Postal 6001, 86051-990 Londrina, PR, Brazil; c Departamento de Química – UFMG, 31270-901 - Belo Horizonte, MG, Brazil

## Abstract

In the title compound, C_6_H_10_N_2_OS, the 2-sulfanylideneimidazolidin-4-one fragment is essentially planar (r.m.s. deviation = 0.0033 Å). In the crystal, one amino group is involved in N—H⋯O hydrogen bonding, which links pairs of mol­ecules into inversion dimers, while the other amino group generates weak N—H⋯S hydrogen bonds, which link these dimers into chains in [10-1]. The chains are further aggregated into layers parallel to the *ac* plane through weak C—H⋯O inter­actions.

## Related literature

For the biological activity of 2-thio­hydantoin derivatives, see: Ghoneim *et al.* (1987 ▶); Marton *et al.* (1993 ▶). For the crystal structures of related compounds, see: Kunimoto *et al.* (2009 ▶); Ogawa *et al.* (2007 ▶). For details of the synthesis, see: Wang *et al.* (2006 ▶).
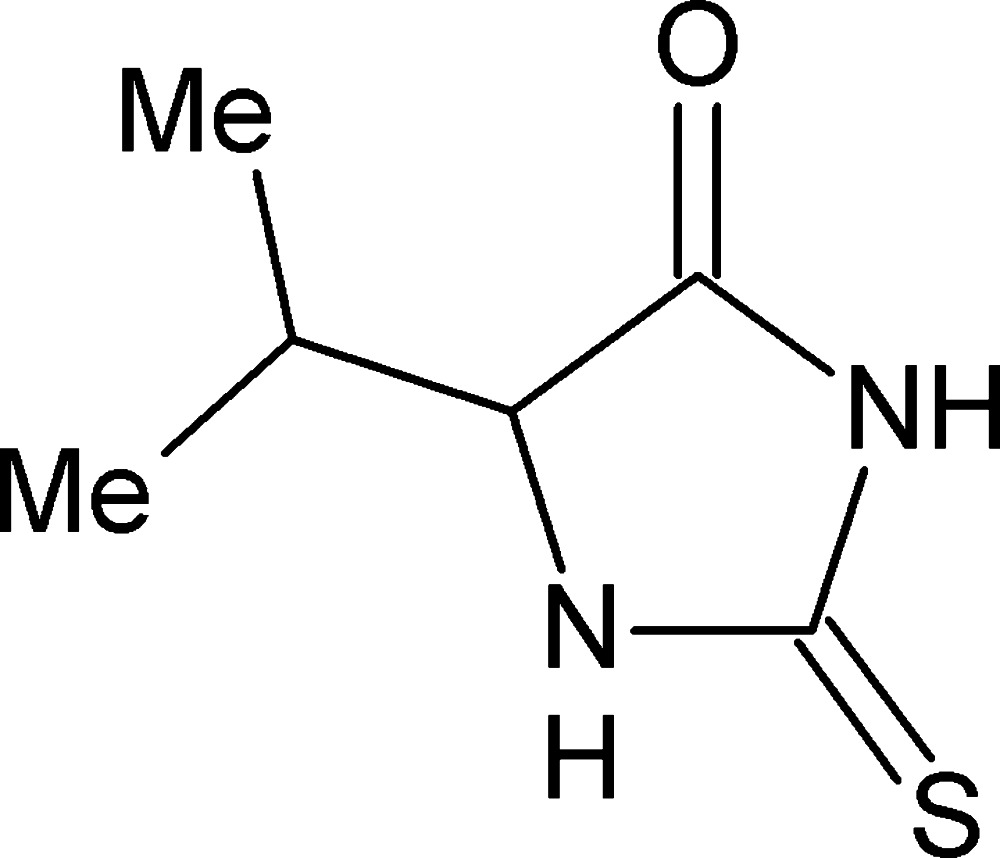


## Experimental

### 

#### Crystal data

C_6_H_10_N_2_OS
*M* *_r_* = 158.23
Monoclinic, 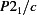

*a* = 5.7161 (1) Å
*b* = 17.4091 (4) Å
*c* = 8.2505 (2) Å
β = 103.513 (1)°
*V* = 798.30 (3) Å^3^
*Z* = 4
Mo *K*α radiation
μ = 0.34 mm^−1^
*T* = 308 K
0.93 × 0.4 × 0.3 mm

#### Data collection

Bruker APEXII CCD diffractometer
Absorption correction: multi-scan (*SADABS*; Bruker, 2010 ▶) *T*_min_ = 0.936, *T*_max_ = 0.979
18741 measured reflections
3064 independent reflections
2544 reflections with *I* > 2σ(*I*)
*R*_int_ = 0.020

#### Refinement

*R*[*F* ^2^ > 2σ(*F* ^2^)] = 0.037
*wR*(*F* ^2^) = 0.114
*S* = 1.02
3064 reflections
93 parameters
H-atom parameters constrained
Δρ_max_ = 0.33 e Å^−3^
Δρ_min_ = −0.20 e Å^−3^

### 

Data collection: *APEX2* (Bruker, 2010 ▶); cell refinement: *SAINT* (Bruker, 2010 ▶); data reduction: *SAINT*; program(s) used to solve structure: *SHELXS97* (Sheldrick, 2008 ▶); program(s) used to refine structure: *SHELXL97* (Sheldrick, 2008 ▶); molecular graphics: *ORTEP-3 for Windows* (Farrugia, 2012 ▶); software used to prepare material for publication: *WinGX* (Farrugia, 2012 ▶).

## Supplementary Material

Crystal structure: contains datablock(s) global, I. DOI: 10.1107/S1600536813008337/cv5395sup1.cif

Structure factors: contains datablock(s) I. DOI: 10.1107/S1600536813008337/cv5395Isup2.hkl

Supplementary material file. DOI: 10.1107/S1600536813008337/cv5395Isup3.cml

Additional supplementary materials:  crystallographic information; 3D view; checkCIF report

## Figures and Tables

**Table 1:** Hydrogen-bond geometry (Å, °)

*D*—H⋯*A*	*D*—H	H⋯*A*	*D*⋯*A*	*D*—H⋯*A*
N2—H2⋯O1^i^	0.86	2.02	2.8573 (11)	164
N1—H1⋯S1^ii^	0.86	2.52	3.3806 (9)	176
C6—H6*C*⋯O1^iii^	0.96	2.52	3.4434 (14)	160

Symmetry codes: (i) 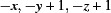; (ii) 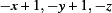; (iii) 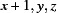.

**Fig. 1.**
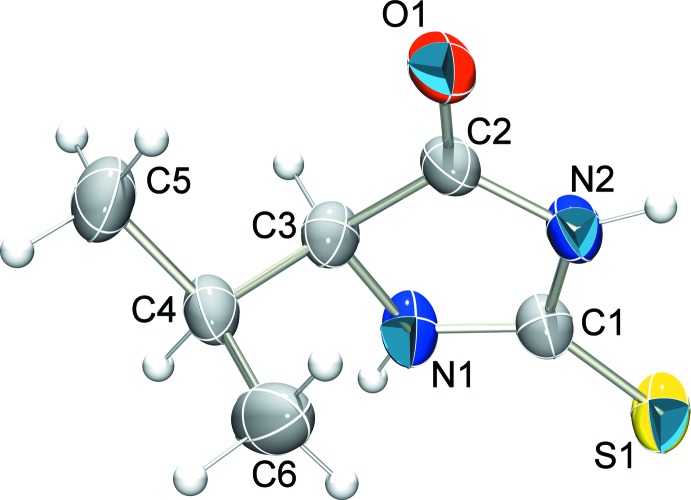
The structure structure of (I) showing the atom-numbering scheme. Displacement ellipsoids are drawn at the 50% probability level

**Fig. 2.**
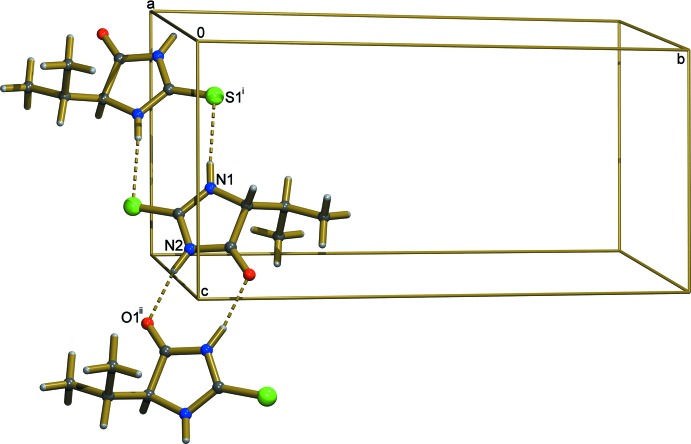
A portion of the crystal packing, showing intermolecular hydrogen bonds as dashed lines [symmetry codes: (i) -x, -y + 1, -z + 1; (ii) -x + 1, -y + 1, -z].

## supplementary crystallographic information

### Comment

2-Thiohydantoin derivatives demonstrate biological activities when used as drugs, fungicides and herbicides (Ghoneim *et al.*, 1987; Marton *et al.*, 1993). Herewith we present the title compound, (I), which is a new 2-thiohydantoin derivative.

In (I) (Fig. 1), the thiohydantoin ring is essentially planar [r.m.s = 0.0033 Å and largest deviation of 0.008 (2) Å for O1]. The orientation of the isopropyl group, defined by the atoms C4, C5 and C6, relative to this plane is given by the torsion angles N1—C3—C4—C5 and N1—C3—C4—C6 of 169.42 (9) and 65.8 (1)°, respectively. The C1—S1 bond, 1.6621 (9), has double-bond character. The N2—C1 bond distance is longer than N1—C1 bond distance by 0.047 Å. The N1—C1—S1 bond angle is greater than N2—C1—S1 bond angle by 3.84°. These differences are also observed in two similar compounds: 5-phenyl-2-sulfanylidene-4-imidazolidinone [CSD refcode: YINGIM (Ogawa *et al.*, 2007)] and rac-5-benzyl-2-thiohydantoin [CSD refcode: KUGDUM (Kunimoto *et al.*, 2009)]. Besides, the molecular geometry of the title compound is comparable to these reference molecules within the standard uncertainties.

Intermolecular N—H···O hydrogen bonds (Table 1) lead to centrosymmetric dimers formation. Weak interactions of type N—H···S (Table 1) mediate the formation of the chains along [101], which are further linked into layers parallel to *ac* plane through the weak C—H···O interactions (Table 1).

### Experimental

The procedure employed for synthesis of compound (I) was described by Wang *et al.* (2006). A mixture of D-valine (2.34 g, 0.2 mol) and thiourea (4.57 g, 0.6 mol) was added in a flask and heated under stirring. The reaction was remained in the oil bath at temperature of 190°C for 30 minutes, after this period, the flask was allowed to cool down and water (20 ml) was added while the flask was still warm. The solution was reheated to dissolve all the solids and allowed to cool to room temperature, then placed in a refrigerator for 3 h. The solid were removed by vacuum filtration and storage. Recrystallization from chloroform yielded single crystals suitable for X-ray analysis.

### Refinement

All H atoms were placed in calculated positions (N—H = 0.86 Å; C—H = 0.96–0.98 Å) and treated as riding atoms, with *U*_iso_(H) = 1.2–1.5 *U*_eq_ of the parent atom.

### Crystal data

**Table:**

C_6_H_10_N_2_OS	*F*(000) = 336
*M**_r_* = 158.23	*D*_x_ = 1.316 Mg m^−^^3^
Monoclinic, *P*2_1_/*c*	Mo *K*α radiation, λ = 0.71073 Å
Hall symbol: -P 2ybc	Cell parameters from 637 reflections
*a* = 5.7161 (1) Å	θ = 7.0–60.8°
*b* = 17.4091 (4) Å	µ = 0.34 mm^−^^1^
*c* = 8.2505 (2) Å	*T* = 308 K
β = 103.513 (1)°	Prism, colourless
*V* = 798.30 (3) Å^3^	0.93 × 0.4 × 0.3 mm
*Z* = 4	

### Data collection

**Table:**

Bruker APEXII CCD diffractometer	2544 reflections with *I* > 2σ(*I*)
Multilayer optics monochromator	*R*_int_ = 0.020
φ and ω scans	θ_max_ = 34.3°, θ_min_ = 2.3°
Absorption correction: multi-scan (*SADABS*; Bruker, 2010)	*h* = −9→8
*T*_min_ = 0.936, *T*_max_ = 0.979	*k* = −26→27
18741 measured reflections	*l* = −12→13
3064 independent reflections	

### Refinement

**Table:**

Refinement on *F*^2^	Primary atom site location: structure-invariant direct methods
Least-squares matrix: full	Secondary atom site location: difference Fourier map
*R*[*F*^2^ > 2σ(*F*^2^)] = 0.037	Hydrogen site location: inferred from neighbouring sites
*wR*(*F*^2^) = 0.114	H-atom parameters constrained
*S* = 1.02	*w* = 1/[σ^2^(*F*_o_^2^) + (0.0671*P*)^2^ + 0.0869*P*] where *P* = (*F*_o_^2^ + 2*F*_c_^2^)/3
3064 reflections	(Δ/σ)_max_ = 0.002
93 parameters	Δρ_max_ = 0.33 e Å^−^^3^
0 restraints	Δρ_min_ = −0.20 e Å^−^^3^

### Special details

**Table:**

Geometry. All s.u.'s (except the s.u. in the dihedral angle between two l.s. planes) are estimated using the full covariance matrix. The cell s.u.'s are taken into account individually in the estimation of s.u.'s in distances, angles and torsion angles; correlations between s.u.'s in cell parameters are only used when they are defined by crystal symmetry. An approximate (isotropic) treatment of cell s.u.'s is used for estimating s.u.'s involving l.s. planes.
Refinement. Refinement of *F*^2^ against ALL reflections. The weighted *R*-factor *wR* and goodness of fit *S* are based on *F*^2^, conventional *R*-factors *R* are based on *F*, with *F* set to zero for negative *F*^2^. The threshold expression of *F*^2^ > 2σ(*F*^2^) is used only for calculating *R*-factors(gt) *etc*. and is not relevant to the choice of reflections for refinement. *R*-factors based on *F*^2^ are statistically about twice as large as those based on *F*, and *R*- factors based on ALL data will be even larger.

### Fractional atomic coordinates and isotropic or equivalent isotropic displacement parameters (Å^2^)

**Table:**

	*x*	*y*	*z*	*U*_iso_*/*U*_eq_	
S1	0.50440 (6)	0.579973 (16)	0.22144 (4)	0.04944 (11)	
C1	0.31293 (17)	0.50804 (5)	0.22060 (11)	0.03529 (18)	
N1	0.25674 (18)	0.45108 (5)	0.11098 (10)	0.0431 (2)	
H1	0.3188	0.446	0.0262	0.052*	
C3	0.07920 (18)	0.39803 (6)	0.14899 (11)	0.03667 (18)	
H3	−0.0682	0.4005	0.0602	0.044*	
C4	0.16814 (19)	0.31474 (5)	0.16973 (13)	0.0415 (2)	
H4	0.2276	0.3015	0.0711	0.05*	
C5	−0.0352 (2)	0.25955 (7)	0.17693 (18)	0.0568 (3)	
H5A	−0.0905	0.2689	0.2764	0.085*	
H5B	0.0219	0.2077	0.1778	0.085*	
H5C	−0.1655	0.2672	0.0811	0.085*	
C6	0.3773 (2)	0.30522 (7)	0.3219 (2)	0.0595 (3)	
H6C	0.5011	0.3417	0.3163	0.089*	
H6B	0.4408	0.2541	0.3236	0.089*	
H6A	0.3215	0.3139	0.4214	0.089*	
C2	0.03374 (17)	0.43453 (5)	0.30588 (12)	0.03636 (18)	
O1	−0.10205 (16)	0.41213 (5)	0.38922 (12)	0.0516 (2)	
N2	0.17754 (15)	0.49851 (5)	0.33699 (11)	0.03987 (18)	
H2	0.1825	0.5291	0.4196	0.048*	

### Atomic displacement parameters (Å^2^)

**Table:**

	*U*^11^	*U*^22^	*U*^33^	*U*^12^	*U*^13^	*U*^23^
S1	0.0669 (2)	0.04239 (16)	0.04620 (17)	−0.02026 (11)	0.02773 (13)	−0.00744 (10)
C1	0.0445 (4)	0.0311 (4)	0.0333 (4)	−0.0024 (3)	0.0153 (3)	0.0008 (3)
N1	0.0626 (5)	0.0386 (4)	0.0348 (4)	−0.0127 (4)	0.0248 (4)	−0.0050 (3)
C3	0.0431 (4)	0.0368 (4)	0.0320 (4)	−0.0061 (3)	0.0126 (3)	−0.0032 (3)
C4	0.0497 (5)	0.0335 (4)	0.0486 (5)	−0.0080 (4)	0.0263 (4)	−0.0118 (4)
C5	0.0625 (7)	0.0448 (6)	0.0676 (7)	−0.0212 (5)	0.0242 (6)	−0.0143 (5)
C6	0.0440 (5)	0.0419 (5)	0.0917 (10)	0.0023 (4)	0.0142 (6)	0.0060 (6)
C2	0.0379 (4)	0.0328 (4)	0.0431 (4)	−0.0009 (3)	0.0190 (3)	−0.0048 (3)
O1	0.0562 (5)	0.0449 (4)	0.0667 (5)	−0.0122 (3)	0.0406 (4)	−0.0155 (4)
N2	0.0486 (4)	0.0345 (4)	0.0431 (4)	−0.0074 (3)	0.0239 (3)	−0.0096 (3)

### Geometric parameters (Å, º)

**Table:**

S1—C1	1.6621 (9)	C4—H4	0.98
C1—N1	1.3300 (12)	C5—H5A	0.96
C1—N2	1.3769 (12)	C5—H5B	0.96
N1—C3	1.4595 (12)	C5—H5C	0.96
N1—H1	0.86	C6—H6C	0.96
C3—C2	1.5179 (13)	C6—H6B	0.96
C3—C4	1.5328 (14)	C6—H6A	0.96
C3—H3	0.98	C2—O1	1.2152 (12)
C4—C5	1.5200 (14)	C2—N2	1.3724 (12)
C4—C6	1.5269 (18)	N2—H2	0.86
			
N1—C1—N2	107.37 (8)	C4—C5—H5A	109.5
N1—C1—S1	128.23 (7)	C4—C5—H5B	109.5
N2—C1—S1	124.39 (7)	H5A—C5—H5B	109.5
C1—N1—C3	113.28 (8)	C4—C5—H5C	109.5
C1—N1—H1	123.4	H5A—C5—H5C	109.5
C3—N1—H1	123.4	H5B—C5—H5C	109.5
N1—C3—C2	100.63 (7)	C4—C6—H6C	109.5
N1—C3—C4	113.17 (8)	C4—C6—H6B	109.5
C2—C3—C4	114.79 (8)	H6C—C6—H6B	109.5
N1—C3—H3	109.3	C4—C6—H6A	109.5
C2—C3—H3	109.3	H6C—C6—H6A	109.5
C4—C3—H3	109.3	H6B—C6—H6A	109.5
C5—C4—C6	111.00 (10)	O1—C2—N2	126.04 (9)
C5—C4—C3	111.44 (9)	O1—C2—C3	127.40 (9)
C6—C4—C3	111.72 (8)	N2—C2—C3	106.55 (8)
C5—C4—H4	107.5	C2—N2—C1	112.15 (8)
C6—C4—H4	107.5	C2—N2—H2	123.9
C3—C4—H4	107.5	C1—N2—H2	123.9
			
N2—C1—N1—C3	−0.87 (12)	N1—C3—C2—O1	−179.83 (11)
S1—C1—N1—C3	−179.62 (7)	C4—C3—C2—O1	−57.99 (15)
C1—N1—C3—C2	0.55 (11)	N1—C3—C2—N2	−0.03 (10)
C1—N1—C3—C4	−122.43 (10)	C4—C3—C2—N2	121.82 (9)
N1—C3—C4—C5	−169.42 (9)	O1—C2—N2—C1	179.33 (11)
C2—C3—C4—C5	75.83 (11)	C3—C2—N2—C1	−0.49 (11)
N1—C3—C4—C6	65.78 (11)	N1—C1—N2—C2	0.84 (12)
C2—C3—C4—C6	−48.97 (12)	S1—C1—N2—C2	179.65 (7)

### Hydrogen-bond geometry (Å, º)

**Table:**

*D*—H···*A*	*D*—H	H···*A*	*D*···*A*	*D*—H···*A*
N2—H2···O1^i^	0.86	2.02	2.8573 (11)	164
N1—H1···S1^ii^	0.86	2.52	3.3806 (9)	176
C6—H6*C*···O1^iii^	0.96	2.52	3.4434 (14)	160

Symmetry codes: (i) −*x*, −*y*+1, −*z*+1; (ii) −*x*+1, −*y*+1, −*z*; (iii) *x*+1, *y*, *z*.
